# Identifying biotic interactions which drive the spatial distribution of a mosquito community

**DOI:** 10.1186/s13071-015-0915-1

**Published:** 2015-07-14

**Authors:** Nick Golding, Miles A Nunn, Bethan V Purse

**Affiliations:** Centre for Ecology & Hydrology, Crowmarsh Gifford, Wallingford UK; Spatial Ecology and Epidemiology Group, Department of Zoology, University of Oxford, Oxford, UK

**Keywords:** Mosquito, Ecology, Species distribution modelling, Ecological interactions

## Abstract

**Background:**

Spatial variation in the risk of many mosquito-borne pathogens is strongly influenced by the distribution of communities of suitable vector mosquitoes. The spatial distributions of such communities have been linked to the abiotic habitat requirements of each constituent mosquito species, but the biotic interactions between mosquitoes and other species are less well understood. Determining which fauna restrict the presence and abundance of key mosquito species in vector communities may identify species which could be employed as natural biological control agents. Whilst biotic interactions have been studied in the laboratory, a lack of appropriate statistical methods has prohibited the identification of key interactions which influence mosquito distributions in the field. Joint species distribution models (JSDMs) have recently been developed to identify biotic interactions influencing the distributions of species from empirical data.

**Methods:**

We apply a JSDM to field data on the spatial distribution of mosquitoes in a UK wetland to identify both abiotic factors and biotic interactions driving the composition of the community.

**Results:**

As expected, mosquito larval distributions in this wetland habitat are strongly driven by environmental covariates including water depth, temperature and oxidation-reduction potential. By factoring out these environmental variables, we are able to identify species (ditch shrimp of the genus *Palaemonetes* and fish) as predators which appear to restrict mosquito distributions.

**Conclusions:**

JSDMs offer vector ecologists a way to identify potentially important biotic interactions influencing the distributions of disease vectors from widely available field data. This information is crucial to understand the likely effects of habitat management for vector control and to identify species with the potential for use in biological control programmes. We provide an R package BayesComm to enable the wider application of these models.

**Electronic supplementary material:**

The online version of this article (doi:10.1186/s13071-015-0915-1) contains supplementary material, which is available to authorized users.

## Background

The spatial distribution of mosquito-borne diseases (MBDs) is dependent on the distribution of suitable vector species [1]. Whilst transmission of some MBDs is entirely dependent on single mosquito species, most MBDs may be transmitted by multiple species [2]. The involvement of multiple vector species with differing biology can complicate vector control interventions. Understanding the ecology and mapping the spatial distribution of these mosquito communities is therefore essential for efficient control of globally important diseases such as malaria [3, 4].

For some zoonotic MBDs, transmission between sylvatic hosts and then to humans requires the presence of multiple species. Such a transmision cycle is exemplified by West Nile virus (WNV). WNV is sustained in an avian sylvatic cycle by ‘maintenance’ mosquito species which must both be ornithophagic and competent for transmission of the virus. Humans and other mammals are ‘dead-end’ hosts for WNV as they rarely develop a sufficient level of viraemia to re-infect mosquitoes [5]. To becoming infected by the virus humans must therefore be bitten by a ‘bridge’ mosquito species which has previously fed on an infected bird and gone on to establish an infection (and therefore must be ornithophagic, anthropophagic and competent). The risk of human WNV cases is therefore restricted to areas where susceptible avian hosts, human populations, maintenance mosquito species and bridge vectors coincide in both space and time.

Unsurprisingly, transmission of WNV in Europe exhibits a patchy distribution which appears to be driven principally by the distribution of its major vectors [6]. In order to identify areas at risk from WNV and similar diseases, both now and in the future, it is therefore crucial to understand what drives the assembly and distribution of entire communities of vector mosquitoes.

Whilst adult female mosquitoes are responsible for transmitting pathogens, the ecology of the relatively immobile larval stages drives the distribution of the species at all but the finest of spatial scales. Herein we refer to the distributions of larval mosquitoes.

### Drivers of community assembly

The majority of previous work on mosquito distributions has focussed on environmental (abiotic) drivers for individual mosquito species [7–9] and mosquito communities [10–13]. Less attention has been paid to the impact of between-species (biotic) interactions on vector distributions. There are a number of ways biotic interaction may influence the distribution of mosquitoes; predation and competition between species may restrict mosquito distributions, whilst apparent or indirect mutualisms may enhance them [14]. Identifying biotic relationships that affect the distributions of vector mosquitoes could be useful for disease control, through habitat management to promote species which control vector mosquitoes [4, 15].

Previous investigations of biotic interactions affecting mosquitoes have mainly been restricted to laboratory experiments [14, 16, 17]. Whilst laboratory studies allow identification of biotic interactions which may be possible under certain environmental conditions, they cannot tell us whether these interactions actually influence the distributions of mosquito communities in the field. Experimental manipulations of communities in the field would provide a fairer test of the impact of specific biotic interactions on the distribution of mosquito communities. Carrying out such experiments at an appropriate spatial and temporal scale and with sufficient replicates to draw generalisable conclusions would likely be costly, extremely difficult to perform and labour intensive particularly, as here, when there are a large number of potential interactions to test.

By contrast, observational data on species co-occurrences are relatively easy to collect. Such data can be interrogated for signs of biotic interactions between species, manifested as correlations between their distributions [18]. This approach is complicated by the difficulty in distinguishing the effects of biotic interactions from environmental factors. Positive (or negative) correlations between species’ distributions could just as easily be explained by sharing (or not sharing) environmental habitat requirements as by the presence of a biotic interaction. To infer biotic interactions from such data, we therefore need appropriate methods to discriminate between biotic and environmental drivers of species distributions.

### Joint species distribution models

Joint species distribution models (JSDMs) have recently been proposed to quantify the effects of biotic interactions on species distributions [19]. Of these, multivariate binomial regression provides an appealing conceptual and technical approach [20]. In such a model each species’ fundamental niche determined by abiotic factors is modelled by independent binomial regressions and biotic interactions between species are modelled as a symmetric matrix controlling correlation in the regression errors between the distributions of different species [21]. Because the model accounts for co-occurrence in distributions which can be explained by each species’ fundamental niche, the positive and negative correlation coefficients are assumed to be representative of positive and negative biotic interactions between species. Parameter inference is carried out using a Markov-Chain Monte Carlo (MCMC) sampler because of the relative complexity of the statistical model.

Applying a multivariate binomial JSDM, we investigate the abiotic environmental factors and inter-species interactions influencing the spatial distribution of a community of potential vector mosquitoes in wetlands in the Thames Estuary, UK. We identify predators of mosquito larvae which appear to influence the distribution of the mosquito vector species and could be managed for vector control. This approach could be applied to rapidly identify candidate species for biological control of vector mosquitoes, whose potential impact could then be assessed by experiments under controlled conditions. We provide an open-source R package to enable vector and community ecologists to apply JSDMs to observational datasets of species distributions.

## Methods

### Larval habitat surveys

**Fig. 1:**
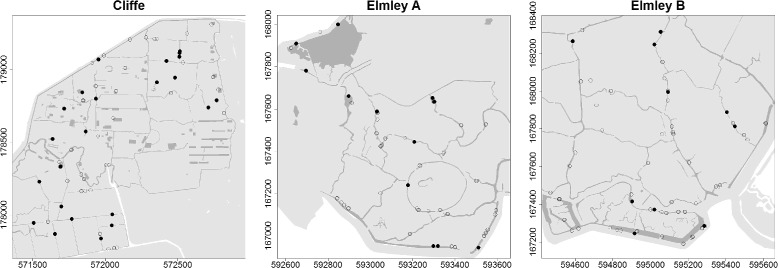
Maps of the three larval survey plots. Filled circles are dip points where mosquitoes were found in at least one dipping round and empty circles where they were not. Land (grazing marsh) is shown in light grey, inland waterbodies (from Ordnance Survey MasterMap) in dark grey and sea in white. Axes are all-numeric Ordnance Survey National Grid references, giving distances in metres

Mosquito larval dipping surveys were carried out at three sites in the North Kent Marshes in south-east England; one at Cliffe marshes (51° 28’ 58” N 0° 28’ 45” E) and two at Elmley Marshes (Elmley A, 51° 22’ 17” N 0° 46’ 27” E and Elmley B, 51° 22’ 29” N 0° 47’ 56” E, see Fig. 1). A set of random geographic coordinates were generated within each site, the nearest water body to each of these points was marked as a dipping site and its location (accurate to within 10cm) recorded using a differential GPS system (Trimble 5800, Trimble Navigation Limited, Sunnyvale, California, USA). A total of 167 dip sites were each visited six times (rounds, taking approximately five days per round); in June, July and August of 2010 and 2011. As few mosquitoes were collected in June of both years, these data were excluded from the analysis. In each round, each dip site was visited and a pair of dips was carried out using a 1 litre dipper; one at the edge and one toward the centre of the water body. The contents of these two dips were pooled for analysis. Mosquito larvae were identified morphologically using taxonomic keys [2, 22, 23] and presence or absence of each species at each dip recorded.

Other ditch fauna were identified morphologically to the most precise level possible in the field [24]. Water temperature, salinity and oxidation-reduction potential were recorded at each dip site and round using a digital probe (YSI 556 MPS, Yellow Springs, Ohio, USA). Water depth was recorded as the mean of the depth at the edge and the centre of each dip site. High-resolution digital photographs (FinePix XP10, Fujifilm, Minato-ku, Japan) were taken of vegetation at the edge and centre dip points and the presence or absence of different vegetation types at each dipsite was determined from these photographs using field guides [25–27].

### Environmental conditions

To identify potential abiotic environmental drivers of distributions and reduce the number of parameters in the JSDM, a forward stepwise selection procedure was applied separately for each faunal taxon to select a subset of abiotic environmental predictors for inclusion in the JSDM. At each step of the procedure, probit regression models were parameterized by maximum likelihood estimation. Starting from a null model, abiotic environmental predictors were added to the model to select combinations of predictors that minimised the model Akaike Information Criterion (AIC) [28]. Terms for dipping rounds and survey area (Elmley or Cliffe Marshes) were included in all of these models to account for the repeated-measures study design. These were modelled as fixed effects as there were insufficient levels (four dipping rounds and three survey areas) to estimate the variance of a random effects term. The full set of environmental covariates from which these subsets were selected consisted of four physical measurements of the waterbody: depth, temperature, oxidation-reduction potential and salinity; and dummy variables indicating presence or absence of the nine vegetation types.

The presence or absence of vegetation types acts as a visible indicator of long-term environmental conditions at dip sites. The plants also strongly influence the structure of the aquatic habitat, variously providing shade or cover from predators. For these reasons we consider vegetation to act as an abiotic environmental condition on mosquito larvae and other aquatic fauna, rather than interacting with them in intimate pair-wise species interactions.

### Joint species distribution model

We use a Bayesian multivariate probit regression model [29, 30] to explicitly model the fundamental niches of each species as well as correlations between the distributions of the different species. Our approach is similar to the model of Ovaskainen et al. [20] but uses the probit function, rather than the logit function as a canonical link. As a result of this modification, we are able to fit the model using a highly efficient Gibbs sampler [31], which greatly speeds up the model fitting process. Full details of the model specification, implementation and choice of priors are provided in Additional file 1. We provide software to fit the model as a free, open-source package BayesComm [32] for the statistical programming environment R. The stable version of the package can be downloaded from the CRAN repositories, or the development version from GitHub at https://github.com/goldingn/BayesComm.

### Effect sizes

As well as identifying environmental conditions associated with the presence or absence of mosquitoes, we wish to compare the strength of these effects in order to pick out the environmental factors which have the most impact on their distribution. Standardizing continuous variables (so that they have a mean of 0 and a standard deviation of 1) enables us to compare the impact of different environmental factors on species distributions without the confounding effects of different measurement scales. The coefficient can therefore be interpreted as the effect of a 1 standard deviation change in the covariate on the distribution of each mosquito species. Since the discrete variables (such as the presence or absence of a vegetation type) are already on the same scale, these can be compared directly as effect sizes. Unfortunately it is not possible to directly compare the effect size of discrete variables with continuous variables.

### Comparing models

As in Ovaskainen et al. we fit four types of model: a *null* model, a *community-only* model, an *environment-only* model and a *full* model. All models were fitted with intercept terms as well as the indicator variables used in the stepwise selection procedure. In the environment-only and full models, for each species the environmental covariates selected by stepwise selection were included in the design matrix. In the null and environment-only models, the correlation matrix was set as an identity matrix, enforcing independence of the model errors between species. In the community-only and full models, the inter-species correlation matrix was also parameterised.

The null model assumes that each taxon occurs with equal probability at each site (conditional on the dipping round and survey area). Any deviation from this prediction can therefore be interpreted as the spatial distribution of the fauna within the study areas. We quantify these distributions as the residual deviance of the null model. By calculating the residual deviance of the other models and the proportion of the null deviance remaining, we measure the proportion of each species’ distribution explained by each model. It should be noted though that since inter-species interactions and environmental covariates are fitted in different ways within the model it is not possible to evaluate the relative importance of the biotic and abiotic factors in driving distributions.

Adding additional parameters to any statistical model inevitably increases its fit to the data, even if there is no true underlying relationship. It is therefore advisable to account for this potential for overfitting when comparing models. A common approach is to use information criteria which penalize models according to the number of parameters added. Our model includes latent variables and some prior information and an error structure and therefore the number of model parameters is not clearly defined. The Deviance Information Criterion (DIC) has been proposed as a natural approach to compare such models [33]. We therefore calculate DIC and use this to compare the likely predictive power of the different models considered here. Lower DICs indicate better fit with a difference greater than 5 indicating an appreciable difference in explanatory power between models.

### Spatial autocorrelation

We checked for a residual spatial autocorrelation structure in each species’ distribution. First we calculated raw residuals using the mean probability of presence at each site as predicted by the full model. We split the dataset by dipping round and survey area, resulting in 8 separate sets of residuals for each taxon (two sites, by two months, by two years). We screened these residuals using a Moran’s I test for spatial independence [34] and for sets with a p-value lower than 0.05 on the I statistic we visually inspected a correlogram for signs of a coherent spatial autocorrelation structure. These univariate spline correlograms were calculated over the full range of distances in each survey area (up to 1700m at Cliffe marshes) with bootsrap resampling. Coherent spatial autocorrelation was defined as the 95 % bootstrap confidence intervals of these correlograms consistently excluding a zero correlation. No such structure was detected in any of the residuals. These analyses were performed using the R packages spdep and ncf [35, 36].

All models were fitted using BayesComm version 0.4 and analysis performed in R version 2.14.2 [37]. The dataset is available via figshare (http://dx.doi.org/10.6084/m9.figshare.1420528) and a full R script to repeat our analysis and reproduce all of the figures in this manuscript is provided in Additional file 2.

## Results

The aquatic faunal community we studied contained 16 taxa, including larvae of four mosquito species - all potential vectors of human disease: *Anopheles maculipennis* s.l. which include *atroparvus* van thiel, *messae* Falleroni and *daciae* Linton, Nicolescu & Harbach in the UK [38], all of which have been detected in the North Kent Marshes [39] and some of which were former vectors of malaria in Europe; *Culex pipiens* s.l. which include *pipiens pipiens* Linnaeus and *pipiens molestus* Forskål, known maintenance vectors of WNV in southern Europe [2]; *Culiseta annulata* Schrank, a potential bridge vector of WNV [40]; and *Culex modestus* Ficalbi, a highly efficient bridge vector of WNV [41–43] only recently found to be breeding in significant numbers in the UK [44] (although a handful of specimens had been reported from around Portsmouth, southern England in the 1944-45 [45]). *Cs. annulata* was only present at Elmley whereas the other three species were present at both study sites. A total of 3,633 mosquito larvae were collected and identified: 1,942 *Cx. pipiens* s.l., 916 *Cx. modestus*, 524 *Cs. annulata* and 251 *An. maculipennis* s.l.

**Fig. 2:**
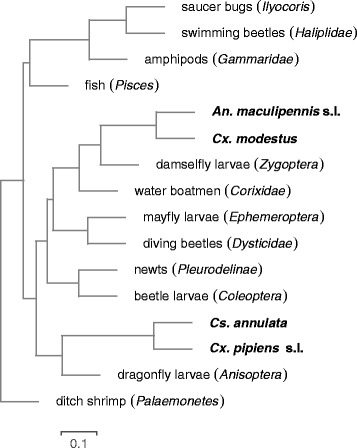
Cluster dendrogram illustrating patterns of co-occurrence between faunal taxa. Taxa closer together in the tree (a short distance from the nodes to their splitting point) were found together more often. The dendrogram was created using hierarchical clustering of the raw distribution data. Mosquito species are in boldface and the scale bar gives the dissimilarity along the edges of the dendrogram. Dissimilarity between two species was calculated as one minus the empirical Pearson correlation coefficient with 0 representing perfect positive correlation, 1 no correlation and 2 perfect negative correlation

Figure 2 illustrates the observed pattern of co-occurrence between the mosquito species and the other recorded fauna. The mosquitoes *An. maculipennis* s.l. and *Cx. modestus* were often found to co-occur, as were *Cs. annulata* and *Cx. pipiens* s.l., though this pair were less often found together. Ditch shrimp were less likely to co-occur with any other species, and a cluster of species including fish, amphipods, swimming beetles and saucer bugs were commonly found together but were less commonly found with other species.

### Environmental drivers

**Fig. 3:**
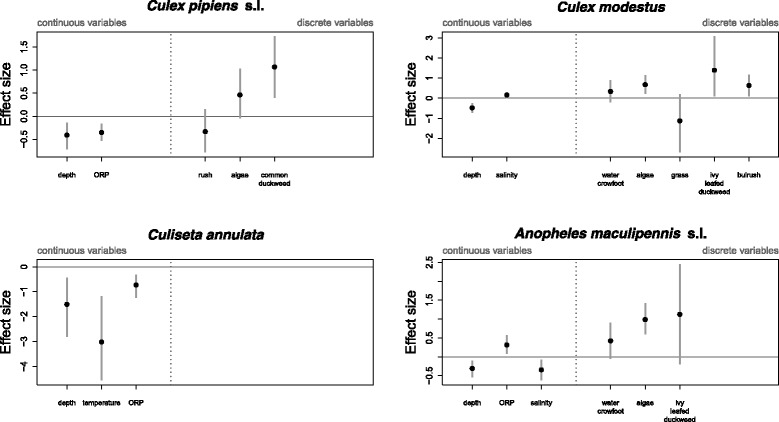
Effect sizes of selected abiotic environmental covariates used to describe the spatial distribution of the four mosquito species estimated using a full model. Points give the maximum *a posteriori* estimates of effect sizes and grey lines give the associated 95 % credible intervals. Credible intervals which do not cross zero signify that the posterior probability that the effect size is 0 or of the opposite sign is less than 0.05, analagous to statistical significance at the 5 % level in a frequentist statistical test. Effect sizes are divided into those for continuous variables (depth, temperature, oxidation-reduction potential (ORP) and salinity) and for discrete variables (plant type), since effect sizes cannot be directly compared for the two different types of data

The relative importance of each of the selected environmental covariates for individual mosquito distributions are displayed in Fig. 3. The main abiotic environmental drivers of the mosquito community distribution were water depth and surface vegetation cover. All species showed a preference for shallow water, with this covariate having the strongest effect on the distribution of *Cs. annulata*. The distributions of *Cx. pipiens* s.l., *Cx. modestus* and *An. maculipennis* s.l. were all positively associated with the presence of surface vegetation (filamentous algae, water crowfoot, and duckweed). The distribution of *Cs. annulata* appeared to be unaffected by the presence or absence of any vegetation type, though it was far more likely to be found in cooler water. Salinity had a slight impact on the distributions of two of the mosquito species, with *Cx. modestus* more likely to occur in more saline water and *An. maculipennis* s.l. in less saline water. Lower oxidation-reduction potential was favoured by *Cx. pipiens* s.l. and *Cs. annulata* and higher oxidation-reduction potential by *An. maculipennis* s.l. Estimates of the environmental regression coefficients for all fauna are given in Additional file 1.

**Fig. 4:**
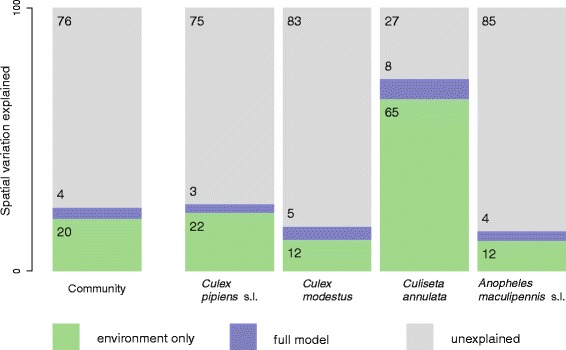
Proportion of spatial variation (measured as deviance) explained by a model with only abiotic environmental covariates and additional variance explained by inclusion of community interactions for the four mosquito species and across the entire mosquito community

Environmental covariates explained 20 % of spatial variation in the distribution of the entire mosquito community (Fig. 4). At the species level, explained variation was 12 % for *Cx. modestus* and *An. maculipennis* s.l., 22 % for *Cx. pipiens* s.l. and 67 % for *Cs. annulata*. Including inter-species correlations in the model accounted for an additional 5 % of spatial variation across the whole community, with an increase of 3 % for *Cx. pipiens* s.l., 4 % for *An. maculipennis* s.l., 5 % for *Cx. modestus* and 9 % for *Cs. annulata*.

### Biotic interactions

**Fig. 5:**
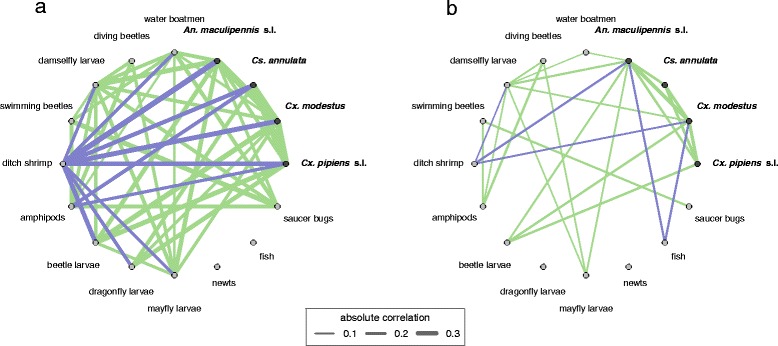
Correlation networks between species in the community **a** before accounting for each species’ fundamental niche (community-only model) and **b** after accounting for fundamental niches (full model). Positive correlations are shown in green and negative in blue. The absolute size of the posterior mean correlation coefficient is reflected in the line width. Correlations are only plotted where the posterior probability of a zero or opposite-sign correlation (Bayesian p-value) was less than 0.05. Parameter estimates used to generate this figure are given in Additional file 1

Estimated inter-species correlation coefficients before and after accounting for environmental covariates are illustrated in Fig. 5. The majority of correlations in the full model were positive (67.5 % from community-only model and 59 % for full model). Correlation coefficients between mosquito species were strongly positive, ranging from 0.28 to 0.49. There were a number of positive correlations between mosquito species and other faunal taxa, including beetle larvae (0.23 to 0.3) and damselfly larvae (0.12 to 0.27). After accounting for each species’ abiotic niche, the distributions of both ditch shrimp (*Palaemonetes*) and fish were negatively correlated with those of the four mosquito species (Fig 5). Correlation coefficients ranged from -0.16 to -0.27 for ditch shrimp and from -0.22 to -0.27 for fish, though the uncertainty around these estimates was larger for *Cx. pipiens* s.l. and *Cs. annulata* than for the other two species (see Additional file 1 for all correlation estimates).

The full model, which contained both environmental covariates and residual inter-species correlations, had greater overall explanatory power (after accounting for model complexity) than any of the other models, with a DIC of 3056 (versus 3648, 3550 and 3150 for the null, community-only and environment-only models).

## Discussion

### Abiotic environmental predictors

The habitat preferences shown by the mosquito species were consistent with existing knowledge of their ecology. All species were more likely to occur in shallower water, with *Cx. pipiens* s.l., *Cx. modestus* and *An. maculipennis* s.l. more likely to occur where floating vegetation was present. Such habitats are likely to afford greater protection from predators [2].

The positive association between *An. maculipennis* s.l. and filamentous algae identified in this study is in accordance with previous studies at Elmley marshes [46] which found the species group was associated with small pools above thick filamentous algae of the genus *Enteromorpha* which were 4 °C warmer, less saline and contained fewer predators than sites without algae. Environmental conditions in this microhabitat appear to be advantageous for mosquito larvae, which are highly susceptible to predation and rely on warm temperatures for their development. Unlike the other members of the mosquito community at these sites, which are Culicine, the morphology of *Anopheles* mosquitoes allows them make use of microhabitats such as these which have limited space at the water-surface interface [2].

The preference of *Cs. annulata* for cooler water and no obvious response to surface vegetation accords with the species’ habitat generalism and common use of shaded water bodies [2]. The strong negative effect of oxidation-reduction potential on the distribution of *Cs. annulata* and *Cx. pipiens* s.l. may reflect their apparent preference for nitrogen rich water [47], though direct interpretation of oxidation-reduction potential alone is difficult.

The negative effects of the presence of emergent grass (indicative of shallow, temporary flooding of normally terrestrial habitat) and deep water (indicative of permanent, open water) on the distribution of *Cx. modestus* may signify a preference for water bodies which are both shallow and relatively permanent. This is supported by the higher probability of presence of *Cx. modestus* in sites with more permanent surface vegetation (water crowfoot, algae and ivy-leafed duckweed). The species was also positively associated with bulrushes, which are known to provide a habitat in which adults of the species hibernate [48, 49].

### Biotic interactions

Most of the non-mosquito fauna we recorded in the community (mayfly larvae being the exception) have been incriminated as potential predators of mosquito larvae in the UK [17]. Wild caught diving beetles, swimming beetles, newts, damselfly larvae and dragonfly larvae have been shown to have consumed larvae [50] and amphipods, ditch shrimp, fish, water boatmen and beetle larvae were shown to feed on live larvae in laboratory experiments [17, 51, 52]. Of these predators, our model provides evidence only for ditch shrimp and fish being negatively correlated with larvae of *An. maculipennis* s.l. and *Cx. modestus* after accounting for environmental covariates. Both ditch shrimp and fish have been found to be particularly voracious predators of mosquito larvae under laboratory conditions, with reports of individuals consuming in the region of 30 larvae (of *Aedes detritus*) per hour, and with ditch shrimp reportedly killing more larvae than they could eat [51]. By comparison, a similar study showed amphipods consumed 1-2 larvae *An. claviger* larvae per hour, and backswimmer (*Notonecta glauca*) nymphs only one larva in 12 hours [52]. The higher predation rate of ditch shrimp and fish in laboratory experiments may explain why they, unlike other predators, have an appreciable impact on the local larval abundance - and therefore the probability of presence of mosquito larvae in seasonal samples in our study system. The identification of fish as effective predators of mosquito larvae is in accordance with findings in other parts of the world, where the introduction of exotic fish or the application of native fish has been successfully used to control mosquito numbers [2].

Whilst the cause of the negative correlation between ditch shrimp and fish and studied mosquito species appears consistent with published literature, it is not immediately clear why this relationship was only apparent for *An. maculipennis* s.l. and *Cx. modestus* in our system and not for *Cx. pipiens* s.l. and *Cs. annulata*. Whilst it is possible that these results are due to intraspecific differences in larval behaviour, the difficulty of replicating natural environments in laboratory predation experiments, and of observing larval behaviour in the wild, means that there is little empirical evidence from which to infer the nature of these interactions for individual mosquito species. It should be noted that the mean correlation coefficients between these two predators and all four mosquito species were negative. However for *An. maculipennis* s.l. and *Cx. modestus* the absolute values of the mean coefficients were smaller than for the other two species and the variance of these estimates was greater (all coefficients given in Additional file 1: Figure 3.2), resulting in a larger Bayesian p-value. The lack of an apparent interaction may therefore simply be a result of insufficient statistical power, rather than a true biological phenomenon. This seems particularly likely to be the case for *Cs. annulata* which was comparatively rare, being present in only 2 % of dip sites (compared with 11-16 % for the other mosquito species).

Of the 11 correlation coefficients between mosquito larvae and other fauna which had good evidentiary support (those in Fig. 5b), 7 were positive and 4 negative. It seems unlikely that such a high proportion of these interactions could represent mutualistic interactions, particularly given the probable predatory nature of the other fauna. A possible explanation is that these positive correlations indicate low-level predation, with the other fauna being attracted to mosquito prey, but not consuming enough to impact on their distribution - at least measured as presence or absence in seasonal snapshot larval sampling where the number individuals sampled is relatively small. A more plausible explanation is that these simply represent shared responses to unmeasured environmental variables which have not been completely explained by the model. This would also explain the strong positive correlations between mosquito species, which might more realistically be expected to compete for resources [16]. Disentangling these competing explanations could be made easier by analysing more temporally-resolved datasets and extending JSDMs to model correlations and lags in the presence and abundance of different species throughout the course of a breeding season, or over several years [19].

### Explanatory power

We considered the distribution of mosquito larvae at a very fine spatial resolution, sampled in brief seasonal snapshots. The presence of mosquito larvae at this scale is likely to be influenced by a number of processes other than those considered here, including: the distribution of blood-meal hosts, local population dynamics and dispersal behaviour of larvae and adults. Despite this, we were able to explain 25 % of the distribution of the mosquito community (and 76 % of the distribution of *Cs. annulata*).

Of the overall variance in spatial variation of the whole community explained by the model, 80 % was due to the effects of environmental drivers and the remaining 20 % to inter-species correlations. Whilst this indicates a much stronger role of abiotic than biotic drivers of mosquito species distributions, it is important to note that these results are scale-dependent - in a smaller study area with lower diversity of larval habitats the relative importance of biotic interactions will be higher, and vice-versa in larger, more habitat-diverse regions. The role of biotic interactions may also be better revealed by sampling regimes that are more intensive both spatially and seasonally. For the individual mosquito species, these relative proportions ranged from 71 % vs. 21 % for *Cx. modestus* to 88 % vs. 12 % for *Cs. annulata*. These results provide information which may be useful for guiding targeted control of mosquitoes. Identifying and promoting species that act as natural biological control agents may be more likely to be effective against mosquito species such as *Cx. modestus* whose distributions appear to be driven to a larger extent by biotic interactions. Conversely, for mosquito species such as *Cs. annulata* which have distributions dominated by availability of particular larval habitat types, targetted removal or treatment of these larval habitats might be more effective.

Whilst the distribution of *Cs. annulata* was very well explained in our models (largely by evironmental covariates), the large parameter estimate for the study area dummy variable in both the environment-only and full models (Fig. 3) indicates that the species’ absence at Cliffe marshes was not explained either by measured environmental covariates or biotic interactions. Why this relatively common species was absent on all visits to this site is as yet unclear.

### Advantages and limitations of joint species distribution models

JSDMs present a promising new method of understanding how interactions within ecological communities can influence species distributions. As with any observational approach to understanding complex systems, JSDMs cannot provide concrete answers to ecological questions. Experimental manipulation of field populations remains the gold-standard for establishing which factors drive species’ and communities’ distributions and for understanding the impacts of habitat modification. However, JSDMs allow the wealth of available observational data on species co-occurrences to be used to refine hypotheses about the drivers of species’ distributions [18].

By combining predictive modelling of the abiotic drivers of individual species’ distributions with a rigorous probabilistic model of species co-occurrence, JSDMs offer significant advances over the classical ordination-type approaches to multivariate analysis of ecological communities [53]. As well as enabling ecologists to begin to disentangle biotic and abiotic effects, JSDMs can more readily be combined with other extensions to linear models, including spatial and temporal correlation and a consideration of phylogenetic structure [54].

## Conclusions

JSDMs are likely to be particularly successful at identifying biotic interactions where abiotic drivers of species’ distributions can be well parameterised and where additional sources of information (such as time series data, commonly available in vector surveys) are available [19].

The relative complexity of JSDMs and large number of model parameters makes fitting such models computationally demanding. As a result, development of JSDMs has only recently become feasible for analysing real ecological datasets. By implementing a computationally efficient sampler for multivariate binomial regression and disseminating it as an R package, we hope to contribute to making these approaches available for routine use by vector ecologists.

## Additional files

Additional file 1
**Further details of the statistical models and inference procedure, and model coefficient estimates.**

Additional file 2
**R script to reproduce analysis and recreate figures in this manuscript.**
