# Supplementary material for: Identifying biotic interactions which drive the spatial distribution of a mosquito community
Source: Parasit Vectors. 2015 Jul 14;8:367. doi: 10.1186/s13071-015-0915-1 (PMC4502600; doi:10.1186/s13071-015-0915-1)
Supplement: Additional file 1 — Further details of the statistical models and inference procedure, and model coefficient estimates. [file 13071_2015_915_MOESM1_ESM.pdf]

Additional file 1 for the manuscript

Identifying biotic interactions which drive the spatial distribution of a mosquito community

Section A - Statistical model and inference

Statistical model

The joint species distribution models (JSDM) we apply here (and which is implemented in the R package we provide `BayesComm`) is specified as a multivariate binomial regression model. We use a multivariate extension of the latent variable model for binary regression (**??**). Our approach is very similar to a model recently described for analysis of ecological communities (**?**) except that we draw the latent variable from a normal, rather than a logistic, distribution. This is equivalent to using a probit function rather than a logit function as the canonical link in a univariate binomial regression. The probit and logit functions are very similar, with the only apparent drawback of the probit model being that regression coefficients for binary covariates can no longer be interpreted directly as log odds ratios (which are not widely used in ecology). The advantage of our approach is that it enables use of a Gibbs sampler to make inference about the regression parameters, thereby reducing computation time and the risk of numerical errors. The model is defined as:

$$\begin{aligned} y_{ij} &= 1(z_{ij} > 0) \\ z_{ij} &= \mu_{ij} + e_{ij} \\ \mu_{ij} &= \mathbf{X}_j \boldsymbol{\beta}_j \\ \mathbf{e}_i &\sim N(\mathbf{0}, \mathbf{R}) \end{aligned} \tag{1.1}$$

where $y_{i,j}$ is a binomial variable representing presence (1) or absence (0) of species j at site i, z is a normally distributed latent variable, $1(z > 0)$ is an indicator function returning 1 when $z > 0$ and 0 otherwise, $\mathbf{X}_j$ is an n by k_j design matrix for species j, $\boldsymbol{\beta}_j$ is a vector of k_j regression coefficients for species j and $N(\mathbf{0}, \mathbf{R})$ is an m-dimensional standard multivariate normal distribution with mean vector $\mathbf{0}$ and symmetric, positive-definite correlation matrix $\mathbf{R}$, n is the number of sites, m the number of species and k_j the number of environmental covariates used to model the fundamental niche of species j. The elements of $\mathbf{R}$ describe whether species co-occur more or less often than would be expected by their fundamental niches alone and is indicative of the underlying network of interactions between species in the community.

Model inference

We use a computationally efficient Gibbs algorithm, based on a sampler described by **?**, to sample the model parameters in turn from their conditional posterior distributions. At each iteration this entails the following steps:

1. Sample the latent variables $\mathbf{z}$ from a truncated multivariate standard normal distribution:

$$\mathbf{z} \sim N_T(\boldsymbol{\mu}, \mathbf{R}) \tag{1.2}$$

such that z_{ij} is positive when $y_{ij} = 1$ and negative otherwise.

2. Sample the vector of regression coefficients $\boldsymbol{\beta}_j$ for each species j from a multivariate normal distribution:

$$\boldsymbol{\beta}_j \sim N((\sigma\mathbf{I} + \mathbf{X}_j'\mathbf{X}_j)^{-1}\mathbf{X}_j'\mathbf{z}_j, (\sigma\mathbf{I} + \mathbf{X}_j'\mathbf{X}_j)^{-1}) \tag{1.3}$$

   where σ is the standard deviation of the prior distribution over $\boldsymbol{\beta}_j$, $\mathbf{I}$ is an identity matrix, having diagonal elements 1 and all other elements 0 and $'$ denotes the matrix transpose.

3. Sample the correlation matrix $\mathbf{R}$ by first sampling a covariance matrix $\mathbf{W}$ and scaling this to a correlation matrix:

$$\begin{aligned} &\mathbf{W}^{-1} \sim \mathcal{W}_m(\nu, \mathbf{e}'\mathbf{e} + \mathbf{S}) \\ &\mathbf{C} = diag(\mathbf{W})^{-\frac{1}{2}} \\ &\mathbf{R} = \mathbf{CWC}' \end{aligned} \tag{1.4}$$

   where $diag(.)$ denotes the diagonal vector of a matrix and $\mathcal{W}_m(., .)$ is a Wishart distribution of dimension m (the number of species). The scale matrix $\mathbf{S}$ and degrees of freedom parameter ν define the prior distribution over $\mathbf{W}$ and therefore $\mathbf{R}$.

By sampling a covariance matrix and scaling to a correlation matrix, we are able to use a Gibbs sampler whilst avoiding the non-identifiability of the variance of a model for binary data. The implications of this approach are discussed in **?**. Our choices of prior parameters σ, $\mathbf{S}$ and ν are discussed in Section 2

Section B - Choice of priors

To construct a sampler for Bayesian statistical models it is necessary to specify priors over all of the parameters for which we want to make inference. These express our prior belief (before observing any data) about the probability distribution of the parameters. Here we use conjugate priors to enable the efficient Gibbs sampler described above.

For each element of each vector of regression coefficients $\boldsymbol{\beta}_j$ we use a diffuse normal prior with mean 0 and variance 100 by setting σ to 10. This is a widely used prior which exhibits little influence on the posterior. Specification of an appropriate prior for the unidentified covariance matrix $\mathbf{W}$ is less straightforward. A commonly used conjugate prior for $\mathbf{W}$ is obtained by setting:

$$\begin{aligned} \nu &= m + 1 \\ \mathbf{S} &= \nu \mathbf{I} \end{aligned} \tag{2.1}$$

This prior has the feature that each element of a correlation matrix derived from $\mathbf{W}$ has a marginally uniform distribution and it therefore has no impact on the posterior. Such a prior is problematic for our model for two reasons. Firstly, a uniform prior implies that it is equally likely for the distributions of two species to be very strongly correlated (i.e. always found together or never found together) as it is for there to be no correlation between them. This is biologically unrealistic; we would expect the majority of inter-species interactions to be weak or non-existent with relatively few interactions driving moderate correlations in distributions (**?**). Secondly, a prior of this sort exhibits a dependency between the unobserved variance parameters of $\mathbf{W}$ and the correlation coefficients of $\mathbf{R}$, such that when these variance parameters are large, the prior assigns much higher probability to strong correlations than weak correlations. This leads to very unrealistic posterior parameter estimates with a bimodal distribution close to 1 and -1. **?** demonstrate a weakly informative prior which avoids this problem but maintains conjugacy for the likelihood:

$$\begin{aligned} \nu &= n + 2m \\ \mathbf{S} &= 2m\mathbf{I} \end{aligned} \tag{2.2}$$

where n is the number of observations. With few observations this gives a reasonable non-uniform prior. As the number of observations increases the prior becomes centred on 0, but with the increased amount of data its influence on the posterior becomes weaker. The shape of this prior and its weak, but informative, effect on the posterior are illustrated using simulated data in Fig. 2.1 and R code to reproduce these figures is given in the supplementary material.

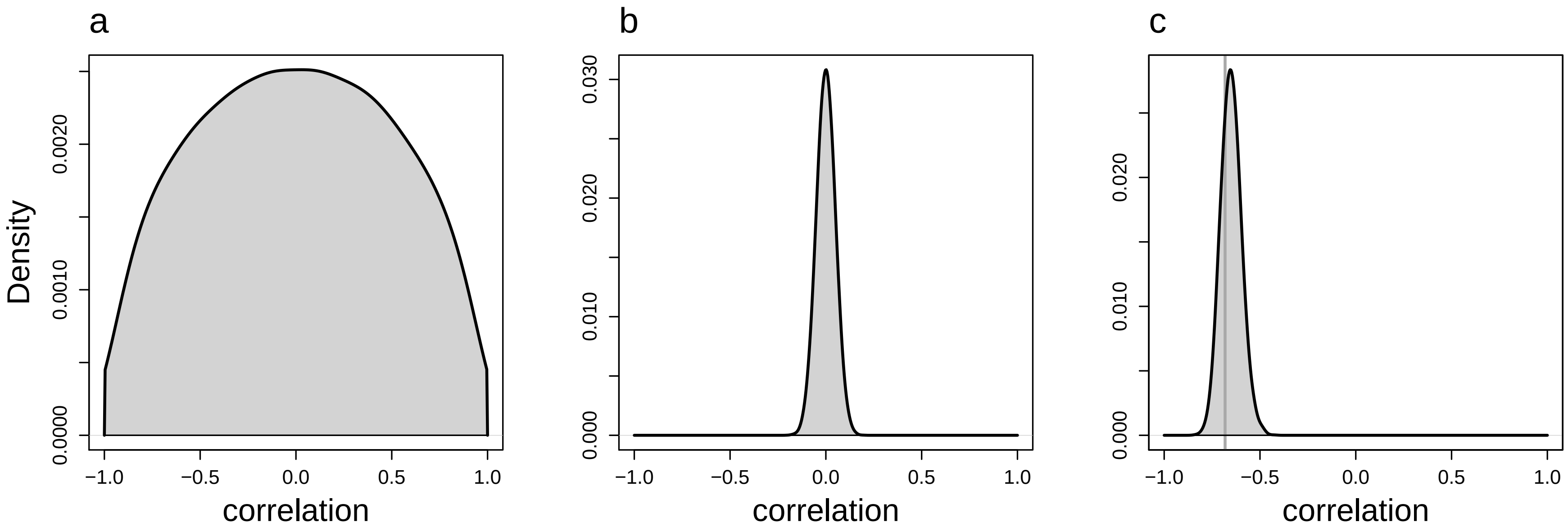

Figure 2.1: Illustration of our inverse Wishart prior over the correlation matrix and its impact on the posteriors. Prior probability density over each element of $\mathbf{R}$ for a) 100 records and b) 400 records, estimated from 100,000 simulations. c) Posterior probability density of the correlation coefficient from a `BayesComm` model applied to a simulated bivariate dataset with 400 observations. The vertical line shows the maximum-likelihood estimate of the coefficient.

Section C - Parameter estimates

The posterior distributions of the regression coefficients from the environment-only and full model are summarised in Fig. 3.1. Summaries of the posterior distributions of the correlation coefficients and the percentage of deviance explained per species from the community-only and the full model are shown in Fig. 3.2.

a

	intercept	depth	temperature	ORP	salinity	water crowfoot (*Ranunculus*)	rushes (*Juncus*/*Scirpus*)	filamentous algae	emergent grass	ivy−leafed duckweed (*Lemna trisulca*)	bulrushes (*Typha*)	reeds (*Phragmites*)	marestail (*Hippuris*)	common duckweed (*Lemna minor*)	August 2010	July 2011	August 2011	Cliffe marshes
Cx. pipiens	−1.18 (0.21)	−0.36 (0.12)		−0.33 (0.08)			−0.38 (0.19)	0.4 (0.23)						0.96 (0.3)	−0.1 (0.2)	−0.74 (0.27)	−0.42 (0.27)	0.07 (0.21)
Cx. modestus	−0.94 (0.14)	−0.43 (0.1)			0.15 (0.07)	0.38 (0.24)		0.57 (0.2)	−1.09 (0.64)	1.3 (0.75)	0.63 (0.23)		0.76 (0.33)		−0.47 (0.17)	−1.27 (0.24)	−0.91 (0.23)	0.15 (0.19)
Cs. annulata	−3.99 (0.7)	−0.77 (0.4)	−1.15 (0.36)	−0.64 (0.16)											−0.03 (0.61)	−0.14 (0.73)	−0.1 (0.98)	−3.25 (1.63)
An. atroparvus	−1.47 (0.16)	−0.26 (0.09)		0.28 (0.12)	−0.35 (0.12)	0.38 (0.23)		0.88 (0.19)		0.98 (0.64)					0.14 (0.2)	−0.55 (0.24)	−0.28 (0.26)	0.11 (0.18)
water boatmen	−0.46 (0.12)	−0.28 (0.06)			−0.23 (0.08)	0.6 (0.2)		0.42 (0.16)		1.35 (0.74)	−0.97 (0.24)	0.33 (0.17)			−0.22 (0.16)	−0.24 (0.16)	−0.55 (0.18)	0.34 (0.15)
diving beetles	−0.92 (0.13)			−0.31 (0.12)											−0.28 (0.18)	−3.63 (1.48)	−3.42 (1.41)	−0.32 (0.2)
damselfly larvae	−1.17 (0.17)					0.6 (0.21)	0.37 (0.15)	0.55 (0.16)	−0.92 (0.63)				0.72 (0.3)		0.33 (0.16)	0.03 (0.16)	−0.05 (0.18)	0.01 (0.14)
swimming beetles	−2.89 (0.39)				−0.27 (0.12)		0.53 (0.24)	0.6 (0.21)	1.29 (0.69)			0.66 (0.25)		0.64 (0.32)	0.6 (0.33)	1.48 (0.3)	1.55 (0.32)	−0.65 (0.22)
ditch shrimp	−0.25 (0.13)			0.41 (0.18)	0.16 (0.06)	−1.31 (0.51)		−1.05 (0.37)			−3.07 (1.45)				0.15 (0.19)	−0.47 (0.24)	−0.47 (0.28)	−1.61 (0.28)
amphipods	−1.04 (0.16)	−0.14 (0.09)		0.25 (0.14)				0.39 (0.25)			−3.17 (1.64)				0.01 (0.22)	0.26 (0.23)	0.16 (0.25)	−1.43 (0.27)
beetle larvae	−3.45 (0.63)	−0.39 (0.17)	0.46 (0.14)				1.42 (0.6)		1.21 (0.66)			−2.71 (1.81)		1.35 (0.49)	−0.55 (0.4)	−0.12 (0.3)	−0.74 (0.47)	0.47 (0.25)
dragonfly larvae	−2.16 (0.32)		−0.32 (0.18)	−0.37 (0.11)											−0.86 (0.46)	0.01 (0.39)	−0.73 (0.58)	−0.06 (0.34)
mayfly larvae	−1.9 (0.28)	−0.31 (0.11)			−0.62 (0.17)		0.43 (0.24)	0.6 (0.22)	−2.7 (1.9)				0.67 (0.39)		−0.13 (0.22)	−0.31 (0.23)	−0.29 (0.29)	−0.39 (0.22)
newts	−5.25 (1.33)	−0.82 (0.38)												1.35 (0.59)	−2.4 (1.31)	−0.07 (0.5)	−0.56 (0.71)	3.04 (1.3)
fish	−2.11 (0.31)	−0.4 (0.18)	−0.38 (0.17)			0.83 (0.34)								−2.99 (2.02)	−1.31 (0.54)	−0.35 (0.39)	−0.51 (0.42)	0.55 (0.29)
saucer bugs	−4.4 (0.92)				−0.3 (0.19)	0.73 (0.31)		1.3 (0.29)			−3.73 (2.08)			1.07 (0.61)	−1.79 (1.63)	2.2 (0.9)	2.62 (0.9)	−0.07 (0.28)

b

	intercept	depth	temperature	ORP	salinity	water crowfoot (*Ranunculus*)	rushes (*Juncus*/*Scirpus*)	filamentous algae	emergent grass	ivy−leafed duckweed (*Lemna trisulca*)	bulrushes (*Typha*)	reeds (*Phragmites*)	marestail (*Hippuris*)	common duckweed (*Lemna minor*)	August 2010	July 2011	August 2011	Cliffe marshes
Cx. pipiens	−1.26 (0.25)	−0.41 (0.15)		−0.35 (0.09)			−0.33 (0.23)	0.5 (0.28)						1.09 (0.35)	−0.17 (0.23)	−0.81 (0.32)	−0.53 (0.34)	0.05 (0.25)
Cx. modestus	−1.01 (0.16)	−0.49 (0.12)			0.15 (0.08)	0.36 (0.28)		0.69 (0.24)	−1.14 (0.76)	1.46 (0.78)	0.63 (0.27)		0.81 (0.38)		−0.53 (0.22)	−1.32 (0.28)	−0.95 (0.28)	0.14 (0.22)
Cs. annulata	−6.96 (0.92)	−1.41 (0.54)	−3.36 (0.64)	−0.83 (0.21)											−0.92 (0.77)	−1.05 (0.81)	−0.91 (1.55)	−3.36 (0.95)
An. atroparvus	−1.56 (0.19)	−0.31 (0.11)		0.32 (0.13)	−0.33 (0.13)	0.42 (0.24)		0.98 (0.2)		1.1 (0.69)					0.15 (0.23)	−0.52 (0.26)	−0.29 (0.3)	0.08 (0.2)
water boatmen	−0.46 (0.13)	−0.28 (0.06)			−0.21 (0.09)	0.6 (0.2)		0.44 (0.17)		1.45 (0.84)	−1 (0.28)	0.32 (0.17)			−0.22 (0.16)	−0.24 (0.16)	−0.56 (0.19)	0.33 (0.15)
diving beetles	−0.9 (0.14)			−0.26 (0.14)											−0.28 (0.2)	−3.51 (1.34)	−2.98 (1.12)	−0.34 (0.22)
damselfly larvae	−1.23 (0.19)					0.62 (0.2)	0.43 (0.16)	0.56 (0.16)	−1.12 (0.71)				0.77 (0.32)		0.34 (0.17)	0.05 (0.18)	−0.05 (0.19)	0 (0.14)
swimming beetles	−3.04 (0.45)				−0.25 (0.14)		0.65 (0.29)	0.6 (0.26)	1.43 (0.74)			0.74 (0.28)		0.78 (0.34)	0.61 (0.37)	1.51 (0.33)	1.57 (0.35)	−0.66 (0.25)
ditch shrimp	−0.26 (0.14)			0.37 (0.2)	0.14 (0.06)	−1.02 (0.51)		−1.11 (0.44)			−2.46 (1.43)				0.17 (0.21)	−0.45 (0.25)	−0.47 (0.3)	−1.59 (0.31)
amphipods	−1.02 (0.17)	−0.19 (0.1)		0.26 (0.17)				0.37 (0.26)			−5.24 (2.52)				−0.03 (0.24)	0.21 (0.25)	0.13 (0.27)	−1.48 (0.31)
beetle larvae	−4.71 (1.11)	−0.53 (0.2)	0.35 (0.16)				2.7 (1.06)		1.62 (0.73)			−0.96 (1.54)		1.45 (0.52)	−0.8 (0.46)	−0.27 (0.34)	−0.99 (0.55)	0.53 (0.27)
dragonfly larvae	−2.13 (0.33)		−0.35 (0.2)	−0.36 (0.11)											−0.98 (0.54)	−0.04 (0.42)	−0.77 (0.66)	−0.09 (0.36)
mayfly larvae	−2.13 (0.33)	−0.39 (0.12)			−0.75 (0.2)		0.55 (0.27)	0.66 (0.23)	−6 (2.95)				0.77 (0.42)		−0.14 (0.24)	−0.29 (0.25)	−0.2 (0.32)	−0.39 (0.24)
newts	−4.99 (1.12)	−1.05 (0.65)												1.51 (0.66)	−2.75 (1.47)	−0.18 (0.56)	−0.59 (0.76)	2.63 (0.91)
fish	−2.21 (0.36)	−0.4 (0.2)	−0.26 (0.17)			0.76 (0.34)								−6.32 (2.58)	−1.12 (0.53)	−0.13 (0.43)	−0.36 (0.45)	0.54 (0.3)
saucer bugs	−4.36 (1.01)				−0.32 (0.22)	0.87 (0.32)		1.31 (0.32)			−7.61 (3.47)			1.38 (0.6)	−2.36 (2.2)	2.05 (0.99)	2.48 (1)	−0.02 (0.33)

Figure 3.1: Regression coefficients from a) the environment-only model and b) the full model. Displayed are the posterior means with standard deviations in parentheses. Positive coefficients are in grey, negative coefficients in dark grey and the size of the text is proportional to their absolute value.

a

	Cx. pipiens	*Cx. modestus*	*Cs. annulata*	*An. atroparvus*	water boatmen	diving beetles	damselfly larvae	swimming beetles	ditch shrimp	amphipods	beetle larvae	dragonfly larvae	mayfly larvae	newts	fish	saucer bugs
Cx. pipiens	4%	0.49	0.5	0.23	0.05	0	0.09	−0.04	−0.31	−0.21	0.34	0.29	0.06	0.08	−0.13	−0.07
Cx. modestus	0.07	3%	0.36	0.48	0.17	−0.02	0.24	−0.02	−0.34	−0.17	0.36	0.21	0.21	0.14	−0.18	0.07
Cs. annulata	0.09	0.11	7%	0.18	0.03	−0.03	0.02	−0.05	−0.3	−0.25	0.22	0.25	0.07	0.07	−0.1	−0.07
An. atroparvus	0.08	0.07	0.12	2%	0.26	0.06	0.35	0.01	−0.37	0.02	0.3	0.06	0.31	0.12	−0.15	0.21
water boatmen	0.08	0.08	0.11	0.07	0%	0.09	0.24	0.19	−0.19	0.12	0.13	0.07	0.14	0.05	−0.01	0.26
diving beetles	0.1	0.1	0.13	0.1	0.09	0%	0.22	0.07	−0.05	0.25	0.13	−0.02	0.22	0.07	0.04	0.14
damselfly larvae	0.08	0.07	0.12	0.07	0.07	0.09	0%	0.14	−0.21	0.22	0.24	0.01	0.25	0.07	−0.02	0.24
swimming beetles	0.11	0.1	0.13	0.1	0.08	0.13	0.09	1%	−0.09	0.29	0.11	0	−0.06	0.02	0	0.34
ditch shrimp	0.09	0.09	0.11	0.09	0.07	0.1	0.08	0.1	1%	0.11	−0.31	−0.22	−0.2	−0.12	0.06	−0.16
amphipods	0.11	0.1	0.13	0.1	0.08	0.1	0.08	0.09	0.09	2%	−0.01	−0.19	0.03	0	0.05	0.29
beetle larvae	0.1	0.09	0.12	0.1	0.1	0.11	0.09	0.12	0.11	0.12	2%	0.11	0.2	0.17	−0.17	0.09
dragonfly larvae	0.11	0.13	0.13	0.14	0.12	0.14	0.12	0.13	0.13	0.14	0.14	0%	0.02	0.02	0.01	−0.06
mayfly larvae	0.1	0.09	0.13	0.08	0.08	0.1	0.08	0.12	0.1	0.1	0.1	0.13	0%	0.06	−0.12	0.12
newts	0.19	0.17	0.19	0.16	0.14	0.14	0.14	0.15	0.16	0.17	0.16	0.17	0.16	0%	−0.09	0.07
fish	0.16	0.15	0.17	0.14	0.11	0.13	0.12	0.14	0.14	0.14	0.14	0.16	0.14	0.16	0%	0.02
saucer bugs	0.14	0.13	0.15	0.11	0.1	0.14	0.1	0.09	0.13	0.11	0.13	0.16	0.12	0.16	0.14	0%

b

	Cx. pipiens	*Cx. modestus*	*Cs. annulata*	*An. atroparvus*	water boatmen	diving beetles	damselfly larvae	swimming beetles	ditch shrimp	amphipods	beetle larvae	dragonfly larvae	mayfly larvae	newts	fish	saucer bugs
Cx. pipiens	26%	0.43	0.35	0.28	0.05	−0.1	0.14	−0.1	−0.16	−0.1	0.3	0.06	0	0.03	−0.22	−0.14
Cx. modestus	0.08	17%	0.37	0.49	0.11	−0.09	0.21	−0.11	−0.23	−0.15	0.28	0.07	0.15	0.12	−0.27	−0.06
Cs. annulata	0.12	0.12	76%	0.3	0.1	−0.13	0.12	−0.08	−0.2	−0.13	0.23	0.02	0.1	0.06	−0.22	−0.07
An. atroparvus	0.09	0.07	0.14	16%	0.15	0	0.27	−0.13	−0.27	−0.02	0.23	0.02	0.2	0.12	−0.25	−0.01
water boatmen	0.09	0.08	0.12	0.08	7%	0.07	0.14	0.1	−0.13	0.08	0.07	0.09	0.05	0.03	−0.09	0.09
diving beetles	0.11	0.09	0.16	0.1	0.09	2%	0.19	0.05	0.08	0.3	0.07	−0.06	0.2	0.03	0.03	0.1
damselfly larvae	0.09	0.08	0.14	0.08	0.07	0.1	5%	0.06	−0.15	0.18	0.11	0.03	0.17	0	−0.07	0.06
swimming beetles	0.12	0.11	0.15	0.11	0.09	0.13	0.09	10%	−0.02	0.25	−0.05	−0.02	−0.16	−0.07	0.03	0.26
ditch shrimp	0.11	0.1	0.15	0.12	0.08	0.1	0.08	0.1	13%	0.08	−0.15	−0.09	−0.04	−0.05	0.12	−0.04
amphipods	0.11	0.11	0.17	0.11	0.08	0.1	0.08	0.09	0.09	10%	−0.05	−0.08	−0.01	−0.05	0.06	0.18
beetle larvae	0.1	0.1	0.14	0.1	0.1	0.13	0.1	0.14	0.13	0.13	28%	0.02	0.08	0.09	−0.2	−0.07
dragonfly larvae	0.15	0.16	0.16	0.16	0.12	0.16	0.14	0.15	0.14	0.17	0.17	20%	0.03	0	0.01	−0.03
mayfly larvae	0.12	0.11	0.15	0.09	0.09	0.11	0.09	0.12	0.11	0.11	0.12	0.14	17%	0.03	−0.12	0.01
newts	0.18	0.19	0.2	0.18	0.13	0.16	0.14	0.17	0.17	0.17	0.17	0.16	0.16	23%	−0.09	0.03
fish	0.14	0.13	0.16	0.13	0.11	0.14	0.12	0.13	0.14	0.14	0.14	0.15	0.14	0.16	14%	0.02
saucer bugs	0.17	0.17	0.2	0.15	0.11	0.16	0.12	0.11	0.15	0.12	0.16	0.16	0.13	0.17	0.15	25%

Figure 3.2: Inter-species correlation coefficients from a) the community-only model and b) the full model. The upper-right triangle gives the mean of the posterior distribution over the correlation coefficients, with positive coefficients in grey and negative coefficients in dark grey. The lower-left triangle gives the standard deviation of the posterior distribution over each coefficient. The diagonal gives the percentage of null deviance explained for each species. In each cell the size of the text is proportional to the absolute value.
